# The impact of partial and complete lockdown during the COVID-19 pandemic on acute surgical services: a retrospective cohort study

**DOI:** 10.1186/s40001-022-00971-x

**Published:** 2023-01-07

**Authors:** Abdel Rahman A. Al manasra, Nadeem B. Alabdallah, Zaid Mesmar, Sami A. Almasarweh, Mohammad Nofal, Osama Shattarah, Qusai Aljarrah, Khalid S. Ibrahim, Doaa Al-qaoud

**Affiliations:** 1 Department of General Surgery and Urology, Faculty of Medicine, Jordan University of Science and Technology, Irbid, Jordan; 2 Princess Basma Teaching Hospital, Ministry of Health, Irbid, Jordan; 3 Jordan University Hospital, University of Jordan, Amman, Jordan; 4 Department of Pediatrics, Faculty of Medicine, The Hashemite University, Zarqa, Jordan

**Keywords:** Surgery, COVID-19, Surgical outcomes, Healthcare

## Abstract

**Purpose:**

The burden of the coronavirus disease of 2019 (COVID-19) pandemic on the healthcare sector has been overwhelming, leading to drastic changes in access to healthcare for the public. We aimed to establish the impact of implemented government partial and complete lockdown policies on the volume of surgical patient admissions at a tertiary referral center during the pandemic.

**Methods:**

A database was retrospectively created from records of patients admitted to the surgical ward through the emergency department. Three 6-week periods were examined: The complete lockdown period (CLP), which included a ban on the use of cars with the exception of health service providers and essential sector workers; A pre-COVID period (PCP) 1 year earlier (no lockdown); and a partial lockdown period (PLP) that involved a comprehensive curfew and implementing social distancing regulations and wear of personal protective equipment (e.g., masks) in public places.

**Results:**

The number of patients admitted to the surgery ward was significantly higher in the PCP cohort compared to the CLP and PLP cohorts (*p* = 0.009), with a 42.1% and 37% decline in patients’ admissions, respectively. Admission rates for patients with biliary pathologies and vascular thrombotic events increased. 30-day mortality rates did not differ significantly between the three periods (*p* = 0.378).

**Conclusions:**

While COVID-19 lockdown regulations had a significant impact on patient admission rates, surgical outcomes were not affected and the standards of care were maintained. Future protocols should strive to improve access to healthcare to avoid complications caused by delayed diagnosis and treatment.

## Introduction

The coronavirus disease of 2019 (COVID-19) pandemic has tremendously affected healthcare systems across the globe. The Jordanian healthcare system being no different, was immediately put under pressure to maintain standards of care across the country while managing the influx of COVID-19 Positive patients into its hospitals [1]. Early in the pandemic, concerns arose that the highly infectious nature of COVID-19 could result in hospital admissions that would overwhelm available ventilators, hospital beds, and other vital medical resources. Protocols were put in place both internationally by the World Health Organization (WHO) [2], as well as nationally by the Ministry of Health (MOH) seeking to prevent the spread of the infection. Social distancing measures that aimed to “flatten the curve” and reduce peak incidence of infection were put in place by governments across the globe, including the Jordanian government. Outpatient Clinics in Jordan were closed or access was limited and all elective surgical interventions were halted until further notice. However, drastic changes in healthcare services provided inevitably occurred, due to the high infectivity of the COVID-19 virus. Hospitals across the globe came under significant pressure as the volume of COVID-19 positive cases increased and intensive care unit (ICU) beds were filled [3, 4]. The impact of lockdown protocols on patient access to healthcare and the mortality and morbidity that follow as a consequence to these protocols has been a concern amongst medical professionals. It has already been demonstrated in the literature that negative lifestyle habits have emerged due to the implementation of lockdowns. There has been a noted reduction in active exercise frequency and duration [5, 6] and a significant increase in calorie intake during lockdown leading to increased weight gain and obesity [7]. The extent of the impact the pandemic had on surgical practice and surgical outcomes, particularly in the long term, is still unclear. Delayed identification and treatment of medical conditions may have long term consequences that are not apparent at the moment. The MOH in Jordan advised all health sectors to cancel all elective surgeries, in anticipation for the influx of COVID-19 patients in need of the hospital beds, as well as a method of prevention through social distancing. Our aim is to investigate the impact of the COVID-19 pandemic and associated lockdown on admissions of general surgical patients at a tertiary hospital that was a referral center for patients during the pandemic in Jordan. We also set out to establish whether complete lockdown measures had better outcomes compared to partial lockdown measures as well as any other positive learning points to be taken from changes in practice during the COVID-19 pandemic, which may aid in the formulation of higher efficiency protocols in the case of a similar public health crisis in the future.

## Materials and methods

### Study design

A database was retrospectively created from patients admitted to the general surgery ward from the emergency department at KAUH. Three periods were examined: the 6-week period after the announcement of the Jordanian government mandated a complete lockdown (15th March 2020 to 30th April 2020), the same period 1 year earlier prior to the pandemic (15th March 2019 to 7th April 2019), and a partial lockdown (1st November to 15th December, 2020).

### *Definitions *[8]

During the complete lockdown period, which lasted for 6 weeks, the lockdown included a ban on the use of cars with the exception of health service providers and essential sector workers. People were allowed to walk to buy groceries from local stores from 10 am until 6 pm. Neighborhoods with confirmed cases were isolated and inter governorate travel was banned. All outpatient services were put on hold except for emergency units. Elective surgeries were postponed until further notice and only emergent/urgent cases were operated on. This lockdown turned into a strictly enforced curfew that was described as one of the world's strictest measures.

In November 2021, Jordan became the Arab country with the highest number of COVID-19-related deaths per capita. Government announced a comprehensive curfew (partial lockdown) that begins at 9 p.m. for businesses and 10 p.m. for individuals and lasts until 6 a.m. the following day. All gyms, public swimming pools, and indoor play areas were announced closed until further notice. The Public Security Directorate issued citations for individuals who failed to observe social distancing regulations and wear personal protective equipment (e.g., masks) in public places, markets, and shops. Similarly, outpatient clinics were closed and only emergent/urgent cases were operated on. Multiple intraoperative measures were also applied to reduce the risk of COVID 19 transmission during surgery, especially minimally invasive surgeries. Based on early recommendations, these measures advised against the use of a laparoscopic approach in operations unless otherwise indicated [9].

### Inclusion criteria

All general surgical patients, in addition to vascular and thoracic surgery patients, aged 18 years or more, and admitted through the emergency unit were included. Data was obtained from the hospital electronic records. Patients with other non-general surgery complaints (e.g., urological, ENT, orthopedic) were excluded. Data on patient age, date of admission and discharge, length of stay, operative and interventional procedures, mortality/morbidity, and final diagnosis upon discharge from hospital were collected. We also collected 30-day follow-up data for the cohort discharged during the COVID-19 pandemic, to establish any adverse events and readmissions in this group of patients.

### Statistical analysis

The number of patients that were admitted to the hospital and underwent interventional procedures along with their length of stay were analyzed for statistical significance. Level of statistical significance was established using the chi-square test/Fisher’s exact test for the number of patients admitted to hospital and surgical intervention, and the Student Standard T test for length of stay. The statistical analysis was two-tailed, and the significance threshold was set at 0.05 or less. The IBM® SPSS® Statistics version 26 was used.

### Ethics

The study protocol was reviewed and approved by the Committee of Research on Human Subjects at the Jordan University of Science and Technology and by the Institutional Review Board at King Abdullah University Hospital. (Non-funded research No: 878-2020). We protected Patients’ confidentiality in accordance with the declaration of Helsinki provisions.

## Results

**Fig. 1:**
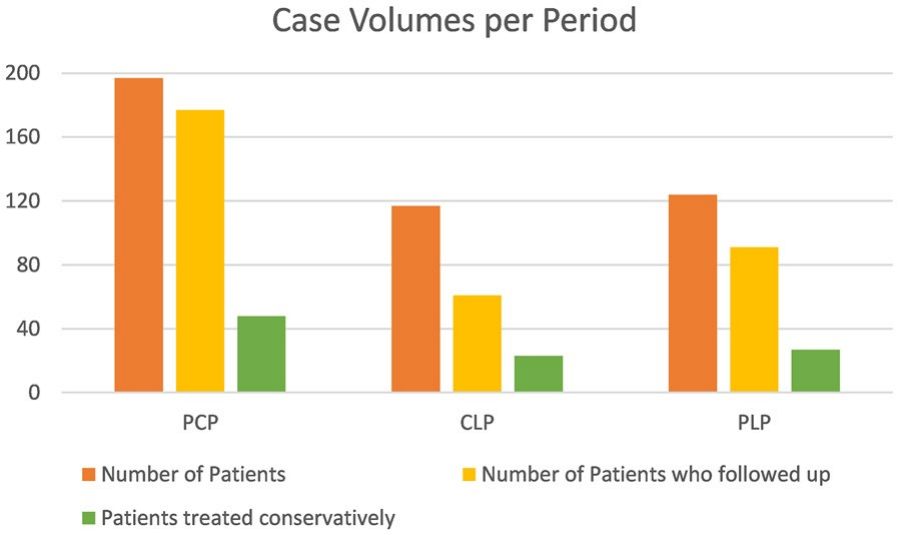
Case volume of admissions during each period

**Fig. 2:**
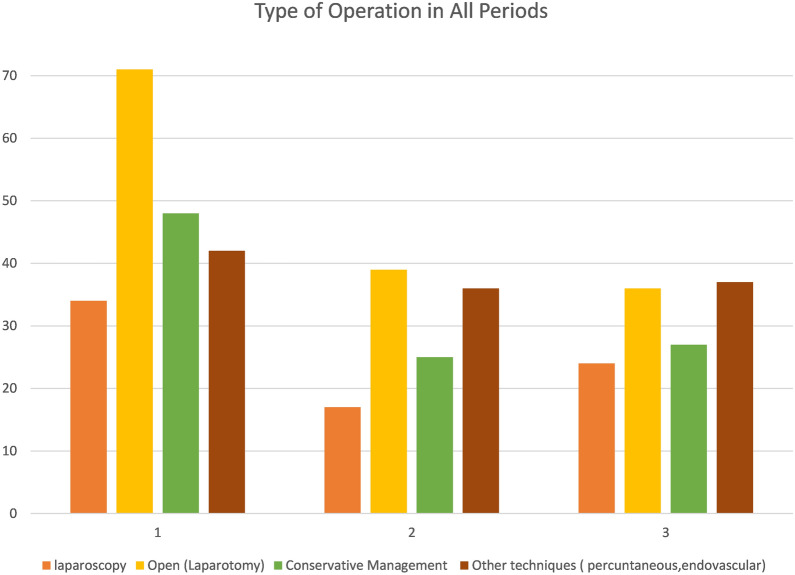
Types of interventions done during each period

Patient data for 436 patients admitted to the surgery ward for operative or conservative management was collected from three different periods: p1 = Pre-COVID Period (PCP), p2 = Complete Lockdown Period (CLP), and p3 = Partial Lockdown Period (PLP). The number of patient data sets for each period was 195, 117, and 124 patients, respectively. A comparison of patient outcomes across the three periods is presented in Table 1. The mean age of the population is 49.4 years with the oldest patient being 90 and the youngest being 1 years. 61.9% of the patients were identified as males and 38.1% as females. The number of patients admitted to the surgery ward was significantly higher in the PCP cohort compared to the CLP and PLP cohorts (*p* = 0.009) (Fig. 1), with a 42.1% and 37% decline in patient admissions, respectively. The distribution of admitted cases for all surgical diagnoses across the three periods is presented in Table 2. All cases followed a similar pattern of decrease in numbers with the exception of patients admitted with Cholecystitis, Obstructive Jaundice, and patients with concomitant End-Stage Renal Disease (ESRD) on hemodialysis. 30-day follow-up was significantly lower during both CLP and PLP compared to the PCP (*p* = 0.000). The number of patients who failed to attend the 30-day follow-up appointment was 11 (5.6%) during the PCP, 49 (41.8%) during the CLP, and 28 (22.5%) during the PLP. Mortality rates did not differ significantly between the three periods (*p* = 0.378). The Mortality rates were 7.6% in the PCP (*n* = 15), 11.9% (*n* = 14) in the CLP, and 7.2% (*n* = 9) in the PLP. COVID-19 PCR testing was performed for 67 patients, five patients during the CLP and 62 during the PLP. Of the 62 patients tested for COVID-19 during the PLP, ten patients (16%) were found to be positive for COVID-19. All five patients tested for COVID-19 during the CLP had negative PCR. Of the ten SARS-CoV-2-positive patients, two patients died. A comparison between operative and conservative management outcomes is presented in Table 3. The difference in the mean length of stay was statistically significant between operative and conservative management (*p* = 0.005). The percentage of patients treated with laparoscopic, open, and conservative approaches was lower in the CLP and PLP cohorts compared to the PCP cohort in favor of percutaneous and endovascular procedures (Fig. 2). However, when vascular surgery procedures were excluded, no difference could be detected (*p* = 0.838).

**Table 1:** Comparison between the three periods

Period	PCP^a^	CLP^a^	PLP^a^	*P* value
Age^b^	48.30 ± 18.95	51.53 ± 17.57	49.21 ± 18.62	0.330
Admissions	195 (44.7%)	117 (26.8%)	124 (28.4%)	0.009
Mortality rate	7 (3.55%)	7 (5.98%)	4 (3.22%)	0.378
30-day follow-up	177 (89.8%)	61 (52.1%)	91 (73.4%)	0.000
Average hospital stay^b^	5.21 ± 5.62	5.99 ± 8.15	5.1 ± 4.53	0.455

^a^*PCP* Pre-COVID period, *CLP* Complete lockdown period, *PLP* Partial lockdown period

^b^Mean ± Standard deviation

**Table 2:** Surgical diagnoses across periods

Diagnosis on discharge	Period			% change in CLP	% change in PLP
	PCP	CLP	PLP		
Acute cholecystitis	15	18	18	20.00%	20.00%
Acute appendicitis	27	1	8	− 96.30%	− 70.40%
Acute pancreatitis	5	0	4	− 100.00%	− 20.00%
Obstructive jaundice	4	7	5	75.00%	25.00%
Intestinal obstruction	8	3	1	− 62.50%	− 87.50%
Strangulated inguinal hernia	3	0	2	− 100.00%	− 33.30%
Femoral hernia	1	0	0	− 100.00%	− 100.00%
Perianal abscess	10	4	3	− 60.00%	− 70.00%
Breast abscess	0	0	2	N/A	N/A
Gluteal abscess	3	0	1	− 100.00%	− 66.70%
Postoperative complications	10	6	1	− 40.00%	− 90.00%
Perforated duodenal ulcer	2	0	1	− 100.00%	− 50.00%
Hemorrhoids	3	0	0	− 100.00%	− 100.00%
Burns	1	1	0	0.00%	− 100.00%
Diverticulitis	1	0	2	− 100.00%	100.00%
Pneumothorax	2	1	1	− 50.00%	− 50.00%
Pneumothorax with lung contusion	1	0	1	− 100.00%	0.00%
Foreign body aspiration	1	0	1	− 100.00%	0.00%
Thoracic empyema	1	0	1	− 100.00%	0.00%
Abdominal aortic aneurysm	3	0	3	− 100.00%	0.00%
Critical lower limb ischemia	28	20	18	− 28.60%	− 35.70%
Toe gangrene	5	5	4	0.00%	− 20.00%
Infected permacath	4	4	1	0.00%	− 75.00%
Infected diabetic foot	9	5	1	− 44.40%	− 88.90%
Cellulitis	3	1	1	− 66.70%	− 66.70%
Infected toe	1	0	0	− 100.00%	− 100.00%
SVC obstruction	1	0	0	− 100.00%	− 100.00%
Carbuncle	1	1	0	0.00%	− 100.00%
AV fistula thrombosis	3	16	11	433.30%	266.70%
Stump wound infection	2	0	0	− 100.00%	− 100.00%
Fournier gangrene	1	0	0	− 100.00%	− 100.00%
Portal vein thrombosis	1	0	1	− 100.00%	0.00%
Wrist cut wound	0	3	0	N/A	N/A
Infected abdominal wound	0	2	0	N/A	N/A
Perineal fistula	0	1	0	N/A	N/A
Common femoral artery thrombosis	0	1	0	N/A	N/A
Sigmoid volvulus	0	0	3	N/A	N/A
Circumferential ulcer	0	0	2	N/A	N/A
others	35	17	27	− 51.40%	− 22.90%
Total	195	117	124	− 40.00%	− 36.40%

**Table 3:** Operative and conservative management comparison

	Operative (*n* = 333)	Conservative (*n* = 98)	*P* value
Age^a^	49.1 ± 18.3	51.5 ± 19.4	0.246
Period			0.665
Pre-COVID Period	147 (44.1%)	48 (49.0%)	
Complete lockdown period	90 (27.0%)	23 (23.5%)	
Partial lockdown period	96 (28.8%)	27 (27.5%)	
Mortality	14 (4.2%)	4 (4.1%)	0.994
Length of stay^a^	5.6 ± 6.6	4.3 ± 2.7	0.005
30-day follow-up	248 (74.5%)	76 (77.6%)	0.841

^a^Mean ± Standard deviation

## Discussion

### Overall admissions

The findings of our study show a significant reduction in the volume of admissions to the surgical ward during periods of COVID-19 lockdown in Jordan. The extent of lockdown was correlated with a drop in the admission volume as predicted, with periods of complete lockdown naturally having lower admission volumes to the surgical ward than periods of partial lockdown. A few studies in the literature reporting on surgical admission numbers during the COVID-19 pandemic were identified, most of which reported a decline in admission volumes [10, 11]. Other studies showing no change in admission rates were also identified [12, 13]; however, these studies focused on trauma and emergency surgical patients in whom urgent surgical attention was required and granted regardless of local and national lockdown policies.

### Gallbladder and biliary interventions

The number of admitted patients who required surgery or invasive interventions for acute Cholecystitis and Obstructive Jaundice increased during the lockdown periods. This can be attributed to the change in policies during the lockdown period, where all elective surgical procedures, including elective cholecystectomies, would be postponed. Furthermore, patients with biliary pathologies might have not been identified altogether early in the disease course, due to outpatient clinics being closed for extended periods of time. This likely allowed for the progression of disease and/or development of obstruction and inflammation within the biliary system. As a result, there was an increase in patients with more severe and complicated Cholecystitis cases that required urgent surgical interventions. This rise in case volume of obstructive jaundice and Cholecystitis patients being admitted to the surgery ward has not been demonstrated in the literature to our knowledge. On the contrary, the results of the recently published international CHOLECOVID study demonstrated over a 30% decrease in cholecystectomy numbers compared to the pre-COVID period. Our results are likely the direct byproduct of lack of access to healthcare and relevant medical expertise for these patients during the pandemic. During CLP, outpatient clinics in Jordan were closed and the only method for transportation to a hospital was by an ambulance in cases of emergency, which led to serious delay in care provided for these patients. This is why we advocate that future government policies consider the long-term patient morbidity associated with these public health measures in mind. We also endorse collaboration with the appropriate medical authorities to formulate policies that maintain the function of the health sector and mitigate complications that result from compromising patient access to healthcare during crises.

### Vascular and hemodialysis access interventions

Patients with End-Stage Renal Disease (ESRD) on Dialysis were another subgroup of patients that had a significant rise in the admission rate to the surgery ward. The number of admissions of ESRD patients increased from one patient during the PCP, to ten and eight patients during the CLP and PLP, respectively. Almost all of these patients were admitted to the surgical ward due to vascular access thrombotic events. It is unclear why ESRD patients were affected this severely by the lockdown, but we hypothesize that the thrombotic events were the result of a multifactorial process involving uncontrolled comorbidities, as patient access to primary care physicians was put on hold, and patients being unwilling to follow-up even when given access to healthcare due to the fear of contracting the COVID-19 virus, which may likely prove fatal in those with compromised immune systems. This theory is supported by previous publications reporting an increase in patients discontinuing their medications during the pandemic, as well as new patients being neither inclined to adhere to nor acquire their medications [14]. Furthermore, COVID-19 has been shown to induce a hypercoagulable state in infected patients [15], which would further increase the risk of vascular access thrombotic events in dialysis patients. A paper by Desbuissons et al. showed that vascular access thrombosis was found to be a severe complication of COVID-19 infection, with a mortality rate of 47% in their study [16]. This high mortality rate is more likely attributed to the COVID-19 infection rather than the thrombotic events, emphasizing the importance of surgical teams becoming acquainted with these complex patient scenarios that they were not used to encountering as frequently prior to the pandemic. This highlights the significance of the interdisciplinary approach and training in providing the best care for these patients and lowering mortality rates. The noted increase in dialysis patients needing admission is also consistent with findings in the literature. A Brazilian multicenter cohort study of 747 dialysis patients found that with the implementation of telemedicine during the pandemic, which was designed to provide patient care without the need for hospital visits, hospitalization rates among dialysis patients actually increased, and the incidence of infections was found to be significantly higher as well [17]. Note that their study looked at all dialysis patients’ admissions, not only surgical ones. Our findings both corroborate and add to their findings, as The increase in patients requiring surgical treatment for AV fistulas may partially reflect the impact on medical outcomes in this subgroup of patients, and calls for more research into the impact of measures like those imposed during the pandemic on their quality of life and morbidity.

### Access to health services

While it stands to reason that social distancing measures are vital to minimize the spread of highly infectious outbreaks, stringent lockdown models appear to be problematic when it comes to patient access to healthcare, resulting in short and long term complications of other patient health issues that are not addressed in a timely fashion. We believe that improved access to health care providers would have reduced the incidence of such complications and, therefore, suggest that future guidelines and lockdown protocols must provide alternative methods of patient care to insure timely diagnosis and treatment of patients. The pandemic has also shed light on the issue of access to healthcare in general, warranting a reevaluation of patient access to healthcare in Jordan, and its social and financial impact on the country as a whole. The introduction of telemedicine in the form of virtual clinics, or portable (mobile) clinics, may prove immensely useful even outside the context of the pandemic, insuring urban as well as rural and underserved communities have adequate medical care, reducing long-term morbidity and mortality.

### Overall morbidity and mortality

We found no significant difference in terms of mortality and length of hospital stay between groups. Other institutions have reported similar findings in the literature as well [11]. This indicates that even with the stringent public health standards employed during the pandemic on national and international levels, the quality of healthcare provided within our health centers can be maintained at a satisfactory level, on par with the standards accomplished prior to the pandemic. Alternatively, it is also possible that the repercussions of the lockdowns have not yet fully materialized and are simply not appreciated. Long term studies aiming to assess the long-term outcomes of the pandemic need to be conducted to improve our understanding of the consequences of the policies implemented during the COVID-19 pandemic, and our understanding of proper resource allocation during disease outbreaks.

### 30-day follow-up

The percentage of patients that presented for a 30-day follow-up during the lockdown periods was significantly lower than the pre-COVID period. These findings are as expected, as private and public transportation was disallowed or limited during the lockdown periods, public fear of contracting the COVID-19 virus was at its highest, and outpatient clinic services were shut down or working at minimal capacity. These factors have led to a decline in patient follow-up rates, although mortality rates were not affected as discussed. Nonetheless, the effects of this decline in follow-up rates may not be appreciated in the short term and future studies should be conducted as stated earlier.

### Open, laparoscopic and percutaneous intervention

While patients managed operatively did not seem to differ substantially from those managed conservatively, except in the duration of hospital stay, the use of laparoscopic, open, endovascular and percutaneous procedures was different across the 3 time periods. Endovascular and percutaneous operations were the only procedures that did not seem to be affected by the lockdown, and thereby comprised a significantly larger proportion of operations undertaken in the lockdown periods compared to the pre-COVID period. This may be due to the urgent nature of vascular procedures when compared to other elective surgeries performed with open technique or laparoscopically. The increase in admission of patients on dialysis also likely correlates with this increase in utilization of endovascular procedures, as most of these patients were admitted with arteriovenous (AV) fistula thrombosis.

This study does have some limitations. First, the study was conducted retrospectively. Second, the number of patients in each group may be too small for the results to be generalized. With that being said, this study was conducted at KAUH, which is an academic tertiary hospital that covers the entire northern region of Jordan and has been designated as a referral center for treatment of COVID cases during the pandemic, which means that this is likely the most representative data available for analysis.

## Conclusions

While COVID-19 lockdown regulations had a significant impact on patient admission rates, patients with biliary pathologies and ESRD patients with vascular access thrombotic events had increased admission rates compared to the prior year, likely due to delayed presentation and the lockdown policies which have interfered with patient access to healthcare service. Surgical outcomes were not affected and the standards of care were maintained. Future protocols should strive to improve access to healthcare to avoid complications caused by delayed diagnosis and treatment. Future lockdown policies should take into consideration the challenges faced by patients requiring medical attention and the long term morbidity and mortality that may be incurred as a result of it. Certain measures should be undertaken to enhance patients’ access to healthcare, especially critically ill individuals, during pandemics and in rural or underserved communities.

## Data Availability

The database created and analyzed during this study is not publicly available as per patient privacy regulations of our hospital but is available from the corresponding author on reasonable request after blinding patient identifying information.
